# Supplementary material for: Tead1a Initiates Transcriptional Priming Through the TEAD1a/YAP‐Notch1‐Spi1/Cebpα Axis to Promote Neutrophil Fate
Source: Adv Sci (Weinh). 2025 Aug 28;12(41):e05441. doi: 10.1002/advs.202505441 (PMC12591127; doi:10.1002/advs.202505441)
Supplement: Supplementary file 4 — Supplementary Dataset [file ADVS-12-e05441-s004.zip › 3-4.zip!/3-4/02-4.cluster/cluster11/Enrich/ego_BP.pdf]

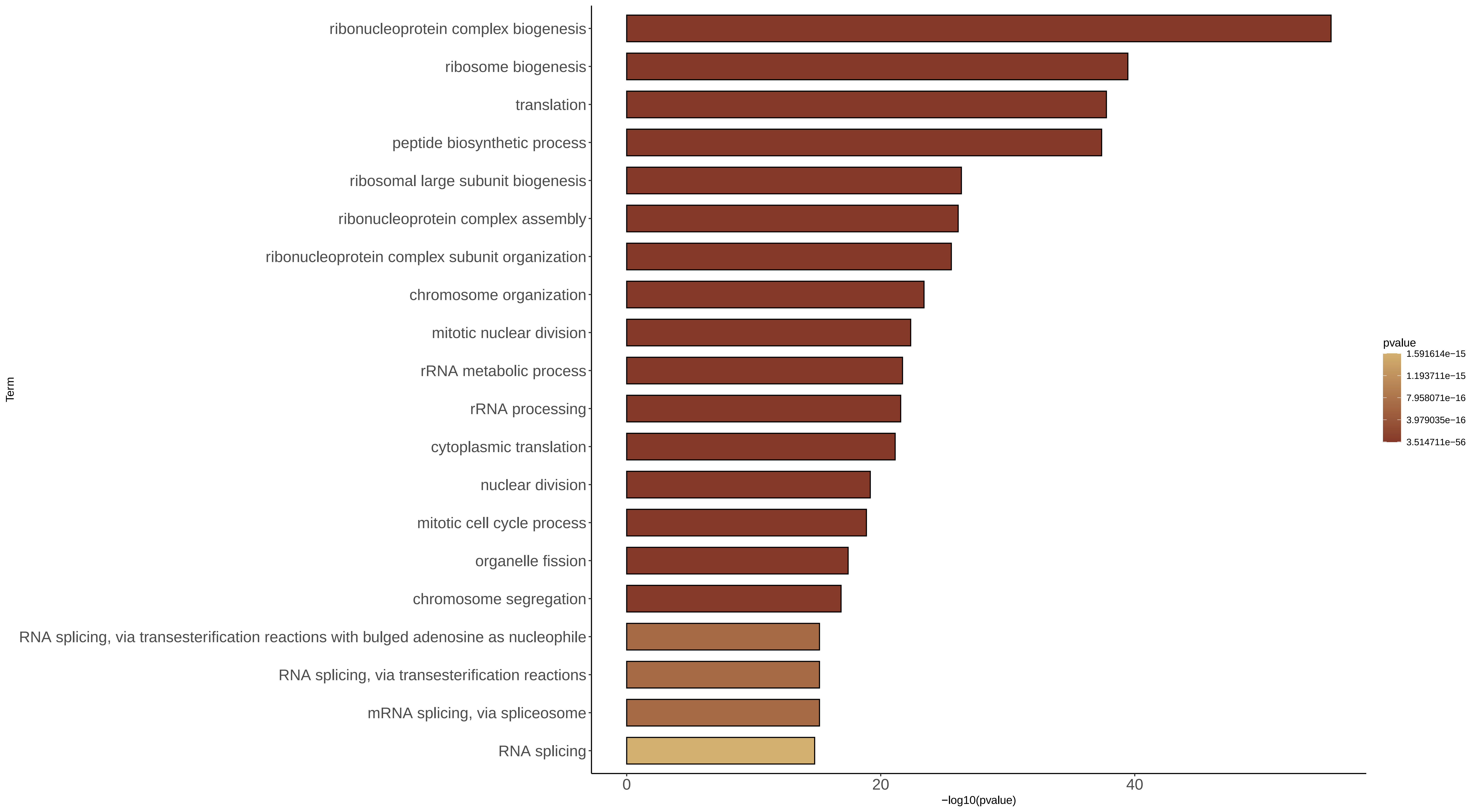

ribonucleoprotein complex biogenesis
ribosome biogenesis
translation
peptide biosynthetic process
ribosomal large subunit biogenesis
ribonucleoprotein complex assembly
ribonucleoprotein complex subunit organization
chromosome organization
mitotic nuclear division
rRNA metabolic process
rRNA processing
cytoplasmic translation
nuclear division
mitotic cell cycle process
organelle fission
chromosome segregation
RNA splicing, via transesterification reactions with bulged adenosine as nucleophile
RNA splicing, via transesterification reactions
mRNA splicing, via spliceosome
RNA splicing
Term
0
20
40
−log10(pvalue)
pvalue
1.591614e−15
1.193711e−15
7.958071e−16
3.979035e−16
3.514711e−56
